# Leveraging large-scale *Mycobacterium tuberculosis* whole genome sequence data to characterise drug-resistant mutations using machine learning and statistical approaches

**DOI:** 10.1038/s41598-024-77947-w

**Published:** 2024-11-07

**Authors:** Siddharth Sanjay Pruthi, Nina Billows, Joseph Thorpe, Susana Campino, Jody E. Phelan, Fady Mohareb, Taane G. Clark

**Affiliations:** 1 Department of Infection Biology, Faculty of Infectious and Tropical Diseases, London School of Hygiene & Tropical Medicine, Keppel Street, London, WC1E 7HT UK; 2 School of Water, Energy and Environment, Cranfield University, Bedford, UK; 3 Faculty of Epidemiology and Population Health, London School of Hygiene & Tropical Medicine, London, WC1E 7HT UK

**Keywords:** Machine learning, Antimicrobial resistance, Bacterial genetics

## Abstract

Tuberculosis disease (TB), caused by *Mycobacterium tuberculosis* (*Mtb*), is a major global public health problem, resulting in > 1 million deaths each year. Drug resistance (DR), including the multi-drug form (MDR-TB), is challenging control of the disease. Whilst many DR mutations in the *Mtb* genome are known, analysis of large datasets generated using whole genome sequencing (WGS) platforms can reveal new variants through the assessment of genotype-phenotype associations. Here, we apply tree-based ensemble methods to a dataset comprised of 35,777 *Mtb* WGS and phenotypic drug-susceptibility test data across first- and second-line drugs. We compare model performance across models trained using mutations in drug-specific regions and genome-wide variants, and find high predictive ability for both first-line (area under ROC curve (AUC); range 88.3–96.5) and second-line (AUC range 84.1–95.4) drugs. To aggregate information from low-frequency variants, we pool mutations by functional impact and observe large improvements in predictive accuracy (e.g., sensitivity: pyrazinamide + 25%; ethionamide + 10%). We further characterise loss-of-function mutations observed in resistant phenotypes, uncovering putative markers of resistance (e.g., *ndh* 293dupG, *Rv3861* 78delC). Finally, we profile the distribution of known DR-associated single nucleotide polymorphisms across discretised minimum inhibitory concentration (MIC) data generated from phenotypic testing (*n* = 12,066), and identify mutations associated with highly resistant phenotypes (e.g., *inhA* − 779G > T and 62T > C). Overall, our work demonstrates that applying machine learning to large-scale WGS data is useful for providing insights into predicting *Mtb* binary drug resistance and MIC phenotypes, thereby potentially assisting diagnosis and treatment decision-making for infection control.

**Supplementary Information:**

The online version contains supplementary material available at 10.1038/s41598-024-77947-w.

## Introduction

Tuberculosis disease (TB), caused by bacteria in the *Mycobacterium tuberculosis Complex* (MTBC), has a high public health burden, with 10.6 million cases and 1.3 million deaths in 2022 alone^1^. However, TB is curable, and with accurate diagnosis and effective treatment, it has the potential to improve disease control outcomes. The standard treatment is a 6-month course of 4 antibiotics, including isoniazid (INH), rifampicin (RIF), ethambutol (EMB), and pyrazinamide (PZA). However, growing resistance to first-line drugs, including RIF (RR-TB) and INH (HR-TB), together called multi-drug resistance (MDR-TB), presents a significant challenge to public health^2^. The development of more severe resistant phenotypes, such as pre-XDR-TB is also a key concern, and is defined by MDR-TB and the additional resistance to a fluoroquinolone, such as ciprofloxacin (CIP), ofloxacin (OFL), or moxifloxacin (MOX). For patients with MDR/RR-TB, the treatment regimen is comprised of bedaquiline (BDQ), prothionamide, linezolid and MOX, and for those who have pre-XDR-TB, the regimen can be used without the fluoroquinolone^3^. Most alarmingly, is the increased prevalence of XDR-TB, which involves pre-XDR-TB and additional resistance to at least one Group A drug (e.g., aminoglycosides, BDQ, linezolid), and further restricts treatment options.

While phenotypic drug-susceptibility tests (pDSTs) and *M. tuberculosis* culture are generally regarded as the gold standard for diagnosis, genome sequencing technologies are increasingly being applied for strain identification and drug resistance prediction, providing valuable insights for clinical decision-making and surveillance activities. The development of resistance in *M. tuberculosis* is associated with point mutations in specific genes, mostly coding for either drug targets or activating enzymes. Putative resistance-conferring mutations against first-line drugs, particularly INH and RIF, have contributed to the development of WHO-endorsed molecular-based diagnostics, which rely on targeted genotype assay methods, such as Genotype MDTBR*plus* (*Hain Lifescience*) or Xpert MTB/RIF (*Cepheid*). Sequencing-based in-silico rule-in classification methods have also been developed and can predict resistance across first- and second-line drugs using thousands of expertly curated drug-resistance mutations (e.g., *TB-Profiler*^4^). However, the full repertoire of mutations for resistance to second-line drugs is unclear, and larger structural variants, such as rare insertions or deletions (indels) and loss of function (LOF) variants, can play an important role in drug-resistance^5^. Further, some mutations (e.g., in *gid* gene) lead to low levels of resistance, which has been observed through differences in odds ratios^6^ and the analysis of minimum inhibitory concentration values (MICs) across drugs^7^.

With the increased application of whole genome sequencing (WGS), combined with the generation of pDST data, to assist TB diagnosis and management, there are opportunities for the application of computational methods to predict drug resistance phenotypes and characterise underlying mutations with greater sophistication^8^. These studies have included the application of statistical techniques, such as genome wide association study (GWAS) based regression approaches^6^, and the development of robust algorithmic approaches to infer drug-specific associations of genomic markers in the presence of co-occurring resistance phenotypes^9^. Machine learning (ML) techniques are particularly well suited for handling high-dimensional data and have demonstrated strong predictive performance in identifying drug resistance from *M. tuberculosis* WGS data. Such approaches are not only capable of predicting resistance, but can also be applied to draw insights into the mutations that hinder drug-susceptibility. For example, interpretable tree-based models have been a popular choice to mine TB genotype-phenotype associations and generate predictions^10–12^. In addition, kernel-based support vector machine (SVM)^13^ and deep-learning methods (Convolution Neural Networks)^14,15^ have also been utilised. Whilst these models can achieve high sensitivity in predictions, they do not always demonstrate a clear improvement in overall predictive performance, as measured by the area under the ROC curve (AUC), and often provide limited interpretability. It can also be difficult to implement published models, but recent ‘container’ based frameworks can be used to build, store, and compare approaches^16^.

Building on this work, our study aims to apply and evaluate the performance of ML models on a large dataset comprised of > 35,000 MTBC with WGS, spanning all major lineages and sourced from over 60 countries. We apply XG*Boost*^17^; a scalable and efficient implementation of Gradient-Boosted-Trees (GBTs), which has demonstrated strong performance across various applications, including TB. We assess the impact of different feature sets on model performance, including drug-specific regions (targeted approach), as well as the inclusion of pooled rare variants and LOF mutations. Furthermore, we use available MIC data and categorise samples by resistance severity, using established epidemiological cut-off (ECOFF) thresholds^18^. We analyse the distribution of known drug resistance mutations across the phenotypic categories to examine genotype-phenotype associations with greater resolution. Further analysis reveals previously unreported putative variants that account for additional variation in resistance phenotypes.

## Results

### *Mycobacterium tuberculosis* genomic diversity and distribution of drug resistance

WGS (> 20-fold coverage) and corresponding pDST data were publicly available for 35,777 *M. tuberculosis* isolates. This “35k” dataset included all four major lineages, with L4 and L2 being the most abundant (L1 9.1%; L2 29.0%; L3 14.1%; L4 46.7%) (Table 1). The isolates were sourced from 66 different countries, covering all WHO regions (Table 1). The availability of pDST data varied by drug. The most complete data was available for first-line drugs RIF (95.0%), INH (93.6%), and EMB (84.7%), while data for PZA was more limited (42.6%). Resistance to Rifabutin (RFB), which is used to treat TB in those who cannot tolerate RIF (e.g., patients with HIV/AIDS), was also analysed. Of the 35k isolates, 43.9% demonstrated phenotypic resistance to at least one of the drugs included, and there was a high prevalence of phenotypic MDR-TB+ (27.6%), pre-XDR+ (16.9%) and XDR-TB (5.3%) (Table 1). Due to TB patients receiving combinations of treatments, the co-occurrence of phenotypic resistance across 13 drugs was assessed (Fig. S1). Evidence of co-occurrence was observed amongst commonly co-administered first-line drugs (RIF, INH, PZA, EMB) (Pearson correlation range 0.58–1) and two second-line classes, such as fluoroquinolones (0.70–1) and aminoglycosides (0.65–1). RFB is a derivative of RIF, which accounts for their strong correlation (0.91). Further, there was a high concordance between genotypic resistance, as inferred by *TB-Profiler, *and pDST, ranging from 89% to 97% (Table S1), particularly for RIF (97%), INH (96.6%), MDR-TB (96.8%), and CIP (97.5%).

**Table 1:** Characteristics of the *M. tuberculosis* dataset (*N* = 35,777).

Characteristics	*N*	%
Lineage		
L4	16,687	46.6
L2	10,365	29.0
L3	5038	14.1
L1	3266	9.1
Others	421	1.1
World Health Organization Region		
Europe	13,381	37.4
Africa	8756	24.5
South East Asia	5518	15.4
Western Pacific	3964	11.1
Americas	3176	8.9
Other	982	2.8
Genotypic drug-resistance		
MDR-TB	433	1.2
Pre XDR-TB	4034	11.3
XDR-TB	2156	6.0
Susceptible	18,378	51.3
Other	10,776	30.2

*MDR* Multidrug-Resistant TB, *Pre-XDR* Pre-Extensively Drug-resistant TB, *XDR* Extensively Drug-resistant TB.

### Applications of machine learning approaches

We evaluated two different ensemble tree-based ML methods - random forest (RF) and gradient boosted trees (GBTs) - assessing their predictive performance (AUC, sensitivity, and specificity) on a hold-out test dataset (Table S2) (see “Methods” for details). There were initially three possible input sets of variants, including in drug-specific regions (total 58 candidate genes; 56–143 variants) (F1), across 58 candidate genes (895–1428 variants) (F2), and genome-wide common variants (5603–10,487 variants) with non-major allele frequency > 0.5% (F3). Across ML methods, we predicted phenotypic resistance for 14 drugs (INH, RIF, EMB, PZA, streptomycin (STM), OFL, MOX, levofloxacin (LEV), amikacin (AMI), capreomycin (CAP), kanamycin (KAN), CIP, ethionamide (ETH) and RFB). Overall, the highest AUC values were achieved using the feature set (F2) that incorporated filtered genomic variants across all candidate genes for each prediction. The combination of GBTs with the F2 feature set (GBT + F2) achieved the highest AUC performance for ETH (86.3), OFL (88.2), PZA (88.3), MOX (89.1), KAN (91.0), RFB (94.9), and CIP (95.4) (Table 2).

**Table 2:** Best-performing models (AUC) on hold-out test dataset for the across 14 drugs.

Drug	*N*	Best model	AUC %	Sens. %	Spec. %	PPV %	NPV %	Accuracy %
INH	33,313	RF + F2	96.50	95.00	98.00	96.80	96.90	96.80
RIF	33,087	RF + F2	96.60	95.50	97.50	94.60	98.00	96.90
EMB	29,950	GBT + F1	91.70	92.00	91.20	70.20	98.00	91.40
PZA	15,107	GBT + F2	88.30	84.44	92.20	71.60	96.20	90.70
STM	11,383	RF + F2	89.60	88.00	91.30	84.00	93.50	90.10
AMK	17,624	RF + F1	90.70	82.30	99.00	90.00	98.20	97.50
CAP	10,215	RF + F2	84.10	71.80	96.50	69.80	96.80	94.00
KAN	18,328	GBT + F2	91.00	83.50	98.68	90.50	97.50	96.70
CIP	402	GBT + F2	95.40	92.30	98.50	92.30	98.50	97.50
OFL	3520	GBT + F2	88.10	81.00	95.30	84.90	93.70	91.80
MOX	15,829	GBT + F2	89.10	84.30	93.90	72.50	96.90	92.40
ETH	14,649	GBT + F2	86.30	81.60	91.00	69.50	95.10	89.00
LEV	15,617	GBT + F1	93.50	88.90	98.10	92.30	97.20	96.30
RFB	10,815	GBT + F2	94.90	93.50	96.30	93.80	96.20	95.30

*AUC* area under curve, *Sens* sensitivity, *Spec* specificity, *PPV* positive predictive value, *NPV* negative predictive value, *F1* variants in drug-specific regions, *F2* variants across candidate genes, *RF* random forest, *GBT* gradient boosted tree, *INH* isoniazid, *RIF* rifampicin, *EMB* ethambuto, *PZA* pyrazinamide, *STM* streptomycin, *AMK* amikacin, *CAP* capreomycin, *KAN* kanamycin, *CIP* ciprofloxacin, *OFL* ofloxacin, *MOX* moxifloxacin, *ETH* ethionamide, *LEV* levofloxacin, *RFB* rifabutin.

In contrast, the combined RF and F2 (RF + F2) approach achieved the highest AUC for INH (96.5), RIF (96.6) and CAP (84.1). Models with the highest AUC generally yielded the highest sensitivity values, with exceptions for EMB (RF + F2, 91.8%), PZA (RF + F2, 85.0%) and LEV (GBT + F2, 92.5%) (Table 2). Whilst most models performed better with the F2 dataset, certain drugs showed highest AUC with the drug-specific candidate gene regions (F1), notably for EMB (GBT, 91.7), AMK (RF, 90.7) and LEV (GBT, 93.5), highlighting the value of a more targeted approach (Table 2). Among RF models, F1 slightly outperformed F2 for MOX in sensitivity (+ 0.5%). Specificity remained high across all models (ranges 89.0–99.5%). Finally, GBT models were applied to genome-wide variants (F3), but this did not significantly improve predictive performance over F2 for any drug (Table S3).

### Inclusion of rare functional variants through aggregating variant counts

We aggregated variant counts per gene for each isolate (see “Methods”) to capture low-frequency mutations (< 0.1%) within coding regions, using them to supplement the F1 feature set (“F1+”). For the GBT models, F1+ yielded improved AUC values compared to F1  for INH (96.0), PZA (89.3), STM (90.9), CAP (86.1), MOX (90.2), ETH (85.0), and LEV (93.8) (Table S4). Considerable improvements in sensitivity were observed for PZA (81.6%), STM (91.3%), CAP (74.3%), and ETH (80.2%), compared to F1. Specificity was comparable across the feature sets (ranges F1 90.3–99.3%; F1+ 91.2–99.5%). Feature importance analysis of these updated models confirmed that the aggregated counts contributed additional information gain, enhancing predictive performance (Figs. S2–S6). For PZA, the highest total information gain was for aggregated *pncA* mutations, with impacts on protein sequence modification and high deleteriousness (Fig. S5).

### Comparisons with other models and rule-in classification

**Fig. 1:**
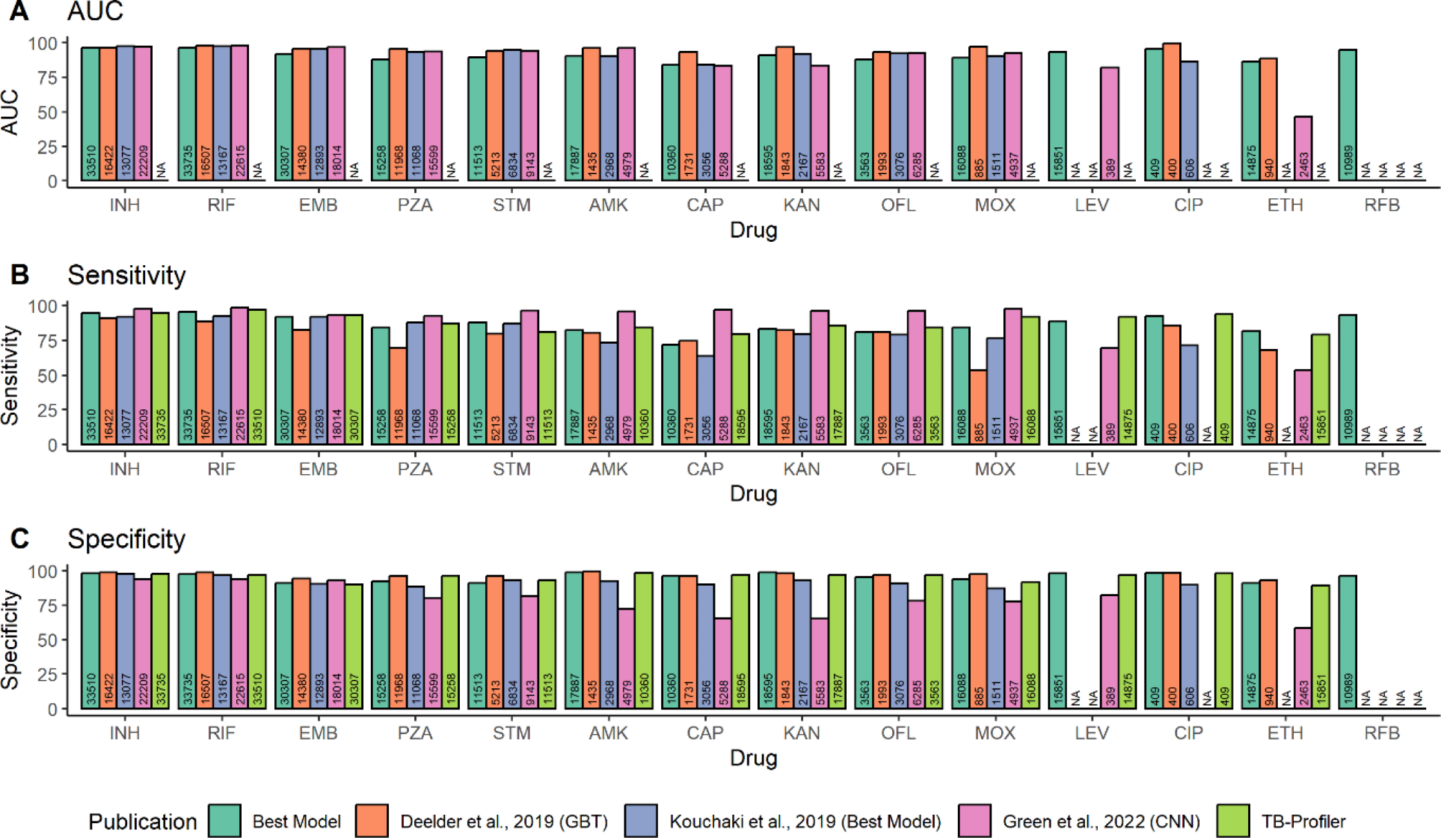
Comparing predictive performance of our ’Best Model’ to models from previously published studies. AUC (**A**), sensitivity (**B**) are specificity (**C**) are compared between our ’best model’ as highlighted in Table 2 to TB-Profiler and previously published models: Deelder et al., 2019 (GBT-CRM), Kouchaki et al., 2019 (Best Ensemble Tree Model) and Green et al., 2022 (CNN). ’NA’ indicates where no samples were available for prediction for that specific drug. Numbers inside bars represent the number of samples available for each drug in each study. TB-Profiler results were made using the samples used in this study. *INH* isoniazid, *RIF* rifampicin, *EMB* ethambutol, *PZA* pyrazinamide, *STM* streptomycin, *AMK* amikacin, *CAP* capreomycin, *KAN* kanamycin, * CIP* ciprofloxacin, *OFL* ofloxacin, *MOX* moxifloxacin, *ETH* ethionamide, *LEV* levofloxacin, *RFB* rifabutin. Low numbers of resistant isolates were available for ethionamide and ciprofloxacin prediction by Green et al., (2022) so performance was not assessed.

We compared the performance of our ML models against other high-performing models (Deelder et al.^10^. (“DE19”), Kouchaki et al.^19^. (“KO19”), Green et al.^15^ (“GR22”); each *n* > 10k), and the in-silico tool TB-Profiler, to assess the benefits of including genome-wide variants and the impact of a larger-scale dataset on predictive performance. Overall, we found comparable performance in terms of AUC, sensitivity, and specificity compared to previously published models (Fig. 1; Table S5). For example, our best-performing (AUC-based) models across the first-line drugs (ranges: AUC 88.3–96.6; sensitivity 84.4–95.5%; specificity 91.2–98.0%) were comparable to DE19 (*n* = 16,507) GBT models (ranges: AUC 95.5–97.9; sensitivity 69.7–91.1%; specificity 94.2–98.9%) (Fig. 1). We observe modest sensitivity improvements for INH (+ 3.9%), RIF (+ 6.7%), EMB (+ 9.2%) and PZA (+ 14.5%). However, our best-performing model showed similar or lower performance for second-line injectables compared to DE19 (ranges AUC 84.1–95.4 for our model vs.  88.4–99.7 for DE19) (Fig. 1). There was an increase in sensitivity for MOX (83.4% vs. DE19 53.5%) and ETH (81.6% vs. DE19 68.1%), likely due to a large increase in sample size (MOX: +15,203 isolates; ETH: +13,935 isolates) (Fig. 1).

We also compared our results with KO19 (*n* = 13,167), who utilised ensemble tree models combined with dimensionality reduction methods on variants in candidate genes, and GR22 (*n* = 22,615), which developed a convolutional neural network (CNN) model. Our best-performing model (ranges 84.1–96.6) had comparable performance across all drugs when compared to KO19 (ranges AUC 84.4–97.8) and GR22 (ranges AUC 82.3–98.1), with the exception of STM (Fig. 1). However, GR22 showed greater sensitivity across all drugs (first-line: 92.8–98.8%; second-line: 65.1–97.2%), except for ETH (+ 27.8%) and LEV (+ 19.5%), where our best-performing model achieved higher sensitivity (Fig. 1). GR22 had limited data on resistant isolates for ethionamide and ciprofloxacin predictions, preventing performance assessment for these drugs within their hold-out data. Interestingly, a larger sample size did not always lead to improved sensitivity. For example, GR22 achieved greater sensitivity (+ 13.8%) with 12,908 fewer samples for AMK (Fig. 1). When comparing our performance to that of the TB*-Profiler* software^4^, we found similar sensitivity values (first-line: 87–97%; second-line: 79.2–92.2%) to ML approaches (Fig. 1, Table S1).

### Association of loss-of-function variants with phenotypic resistance

The assessment of feature importance statistics indicated that aggregated low-frequency mutations play an important role in drug-resistance prediction. To gain further insights, we conducted a targeted association analysis focusing on these low-frequency mutations, particularly loss-of-function (LOF) variants. This analysis revealed 64 distinct significant variants (adjusted *P* < 0.05), with notable associations for ETH (21), INH (17), STM (7), and PZA (6), primarily comprising indels that caused frameshifts (fs) in the gene reading frame (see Data S1 for a full list). Importantly, only a few variants were observed exclusively in resistant isolates, including *pncA* Glu173fs (PZA; odds ratio = 182, L2.2 and L4.3), *ethA* 440_441insT (ETH; OR = 27.6, L2), and *ndh* 293dupG (INH; OR = 12.6, L4.1) (Table 3). Half of the LOF variants (32/64) were found in a single lineage; therefore, we prioritised those in multiple lineages (*n* = 32) (Table 3). Single LOF mutations associated with drug resistance included those linked to BDQ (*Rv0678* Glu49fs, OR = 27.4, *P* < 10^− 6^), CAP (*tlyA* Arg133fs, OR = 26.9, *P* = 2.4 × 10^− 5^), EMB (*embR* Thr120fs OR = 3.3, *P* = 0.0011) and STM (*Rv3861* Ile27fs, OR = 7.0, *P* = 0.002). We also found multiple mutations associated with INH (*ndh* Val238fs; *mshA* Val238fs; *Rv1907c* Asp48fs; *Rv27252c* Arg391fs, Arg97fs, and Gly56fs; ORs > 3), RIF (*Rv27252c* Arg391fs and Arg97fs; ORs > 4.3), PZA (*pncA* Glu173fs, Asp136fs, and Ser65fs; ORs > 12) and clofazimine (CLF) (*Rv0678* Asp47fs and Glu49fs; ORs > 12) (Table 3). Notably, nearly half (15/32) of the prioritised LOF variants were found in *ethA*, which were linked to ETH resistance, including Phe414fs, Asp464fs, Leu225fs, and Pro160fs, with all ORs greater than 10 (Table 3).  We also identified instances of cross-resistance; for example, BDQ and CLF were both associated with LOF variants in *Rv0678*, including Glu49fs (ORs > 12). Similarly, INH and RIF were associated with mutations in *Rv2752c* (Arg391fs, Arg97fs) mutations (Table 3). Most of the prioritised variants (30/32) are in a WHO mutation catalogue^20^, except *Rv1907c* Asp48fs (INH, OR = 4.0, *P* < 10^− 6^) and *Rv3861* Ile27fs (STM, OR = 7.0, *P* = 0.002) (Table 3).

**Table 3:** Loss-of-Function Variants associated with drug-resistant phenotypes.

Drug	Gene	Position	Variant	Conditional OR	*P*-value*	Percent of resistant strains with LOF mutation** (Susceptible %)	Lineage distribution (%)
BDQ	*Rv0678*	779,130	Glu49fs	27.37	< 4 × 10^− 8^	6.8% (0.26%)	2.2 (57.1%), 3 (2.4%), 4.1 (7.1%), 4.2 (2.4%), 4.3 (21.4%), 4.4 (9.5%)
CAP	*tlyA*	1,918,335	Arg133fs	26.90	2 × 10^− 5^	0.58% (0.02%)	2.2 (42.9%), 3.1 (14.3%), 4.3 (28.6%), 4.8 (14.3%)
CLF	*Rv0678*	779,130	Glu49fs	12.72	< 9 × 10^− 9^	2.5% (0.2%)	2.2 (57.1%), 3 (2.4%), 4.1 (7.1%), 4.2 (2.4%), 4.3 (21.4%), 4.4 (9.5%)
		779,127	Asp47fs	12.57	4 × 10^− 5^	1.26% (0.1%)	2.2 (66.6%),3 (5.6%), 4.1 (5.6%), 4.3 (22.2%)
EMB	*embR*	1,416,989	Thr120fs	3.30	0.001	0.26% (0.08%)	1.1 (10.8%),1.2 (2.7%), 2.2 (73.0%), 4.2 (2.70%), 4.4 (8.1%), 4.6 (2.7%)
ETH	*ethA*	4,326,231	Phe414fs	20.66	< 6 × 10^− 13^	0.88% (0.04%)	1.1 (1.8%), 2.2 (63.6%), 3 (7.3%), 3.1 (1.8%), 4.1 (12.7%), 4.4 (1.8%) 4.8 (10.9%)
		4,326,082	Asp464fs	15.80	2 × 10^− 4^	0.27% (0.02%)	2.2 (25.4%), 4.1 (1.82%), 4.8 (72.73%)
		4,326,800	Leu225fs	10.53	5 × 10^− 4^	0.27% (0.03%)	2.2 (46.7%), 3 (6.7%), 4.1 (6.7%), 4.3 (26.7%), 4.8 (13.3%)
		4,326,994	Pro160fs	10.28	1 × 10^− 5^	0.44% (0.04%)	2.2 (90.1%), 4.4 (6.1%), 4.9 (3.0%)
		4,327,472	ethA_c.2T > G	8.48	6 × 10^− 6^	0.51% (0.06%)	2.2 (31.7%), 3 (0.8%), 4.1 (5.8%), 4.2 (60.0%), 6.1 (1.7%)
		4,326,419	Ala352fs	8.41	2 × 10^− 6^	0.57% (0.07%)	1.1 (8.9%), 2.1 (2.2%), 2.2 (46.7%), 3 (6.7%), 4.1 (4.4%), 4.2 (24.4%), 4.4 (2.2%), 5.1 (4.4%)
		4,327,293	Tyr60fs	7.89	0.006	0.2% (0.03%)	2.2 (80%), 4.4 (10.0%), 4.6 (10.0%)
		4,326,439	Asn345fs	5.92	0.009	0.2% (0.03%)	1.1 (81.8%), 4.3 (18.2%)
		4,326,648	Tyr276fs	5.92	0.009	0.2% (0.03%)	2.2 (86.4%), 3 (4.55%), 4.1 (9.09%)
		4,327,363	Lys37fs	4.80	< 6 × 10^− 1^	0.54% (0.12%)	2.2 (94.7%), 3 (0.6%), 4.1 (1.2%), 4.3 (0.6%), 4.6 (2.9%)
		4,326,770	Tyr235fs	4.74	0.014	0.2% (0.04%)	2.2 (96.1%), 3 (3.9%)
		4,326,718	Cys253fs	3.95	0.014	0.24% (0.06%)	2.2 (14.3%), 3 (14.3%), 4 (2.9%), 4.1 (17.1%), 4.3 (28.6%), 4.6 (5.7%), 4.8 (17.1%)
		4,326,087	Arg463fs	3.64	< 6 × 10^− 8^	1.42% (0.39%)	2.2 (98.5%), 3.1 (1.5%)
		4,326,426	Phe349fs	3.55	0.009	0.3% (0.09%)	1.1 (2.1%), 2.2 (32.6%), 3 (15.2%), 4.1 (10.9%), 4.3 (15.2%), 4.6 (8.7%), 4.8 (10.9%), 4.9 (4.4%)
		4,326,589	Leu295fs	3.38	0.033	0.2% (0.06%)	2.2 (94.1%), 4.3 (5.9%)
INH	*mshA*	576,057	Val238fs	9.49	0.018	0.05% (0%)	2.2 (16.6%), 3 (50.0%), 4.1 (16.7%), 4.4 (16.7%)
	*ndh*	2,102,072	Ala324fs	7.91	0.005	0.08% (0.01%)	2.2 (50.0%), 3 (8.3%), 4.1 (16.7%), 4.3 (16.7%), 4.9 (8.3%)
	*Rv2752c*	3,065,022	Arg391fs	9.49	0.018	0.05% (0%)	2.2 (80.0%), 4.8 (20.0%)
		3,065,903	Arg97fs	5.54	0.035	0.05% (0.01%)	2.2 (42.8%), 3 (42.9%), 4.1 (14.3%)
		3,066,026	Gly56fs	4.75	0.018	0.07% (0.01%)	1.2 (9.0%), 2.2 (18.2%), 3 (18.2%), 4.1 (45.5%), 4.4 (9.1%)
	*Rv1907c*	2,153,725	Asp48fs***	4.03	< 10^− 30^	46.61% (17.81%)	1.1 (< 0.1%), 1.2 (0.1%), 2.1 (1.4%), 2.2 (97.9%), 3 (0.1%), 3.1 (< 0.1%), 4.1 (< 0.1%), 4.2 (0.1%), 4.3 (< 0.1%), 4.4 (< 0.1%), 4.5 (< 0.1%), 4.8 (< 0.1%), 4.9 (< 0.1%), 5.1 (< 0.1%)
PZA	*pncA*	2,288,724	Glu173fs	182.28	< 3 × 10^− 29^	1.47% (0%)	2.2 (99.3%), 4.3 (0.7%)
		2,289,049	Ser65fs	15.00	3 × 10^− 4^	0.24% (0.02%)	2.2 (17.8%), 3.1 (64.3%), 4.1 (10.7%), 4.2 (3.6%), 6.3 (3.6%)
		2,288,834	Asp136fs	12.86	9 × 10^− 4^	0.21% (0.02%)	2.2 (48.2%), 4.1 (3.7%), 4.2 (7.4%), 4.3 (37.0%), 4.8 (3.7%)
RIF	*Rv2752c*	3,065,022	Arg391fs	12.92	0.006	0.06% (0%)	2.2 (80.0%), 4.8 (20.0%)
		3,065,903	Arg97fs	4.31	0.033	0.06% (0.01%)	2.2 (42.8%), 3 (42.9%), 4.1 (14.3%)
STM	*Rv3861*	4,338,020	Ile27fs***	7.04	0.002	0.28% (0.04%)	1.1 (6.7%), 2.2 (73.3%), 4.1 (20.0%)

*OR* odds ratio; *adjusted P-value; **percentage of resistant (and susceptible) samples with LOF mutation; ***absent from the WHO catalogue; *OR* odds ratio, *LOF* loss of function, *BQD* bedaquiline, *CAP* capreomycin, *CLF* clofazimine, *EMB* ethambuto, *ETH* ethionamide, *INH* isoniazid, *PZA* pyrazinamide, *RIF* rifampicin, *STM* streptomycin.

### Genomic associations with MIC phenotypes

To gain insights into the levels of resistance conferred by mutations in known target genes, we fitted multinomial ordinal regression models on MIC phenotype data^18^ (see “Methods”). Notably, the variants *Rv1313c* -3741T > C (relative risks (RRs): AMK 10.2, KAN 50.2; adjusted *P* < 10^− 6^) and *gyrA* 280G > A (RRs: LEV 15.6, MOX 9.2; adjusted *P* < 10^− 6^) were frequently identified with the highest risk of resistance among the second-line injectables and fluoroquinolones, respectively (Table 4; see Data S1 for a full list). Additionally, multiple single nucleotide polymorphisms (SNPs) within the short genomic region 1,673,423–1,673,432, which are upstream modifiers of *inhA* and *fabG1*, were identified as being associated with extreme drug resistance against INH ( ≥ ~ 120 x ECOFF MIC threshold ). We also analysed the frequencies of these mutations across MIC categories, which further supports the observed high levels of resistance (Figures S7–S10).

**Table 4:** Summary of the variants with the highest relative-risk ratios for high MIC across 8 drugs.

Drug	Position (gene)	Variant*	Suscept. Coeff.	Low Coeff.	Resist. Coeff.	Resist. *P*-value	Resist. RR
RIF	763,555 (*rpoB*)	230 C > T	− 0.358	0.474	1.913	< 3 × 10^− 7^	6.773
INH	1,673,423 (*inhA*)	− 779G > T**	− 4.843	1.050	4.192	< 10^− 6^	66.168
EMB	4,248,002 (*embB*)	1489 C > A	− 4.584	− 1.930	2.164	< 3 × 10^− 10^	8.707
AMK	1,473,246 (*Rv1313c*)	− 3741T > C **	− 6.422	− 4.975	2.320	< 2 × 10^− 14^	10.174
KAN	1,473,246 (*Rv1313c*)	− 3741T > C **	− 3.684	− 3.063	3.915	< 10^− 6^	50.165
ETH	1,674,263 (*inhA*)	62T > C	− 14.794	0.380	3.457	< 2 × 10^− 6^	31.708
LEV	7581 (*gyrA*)	280G > A	− 2.933	− 3.774	2.746	< 10^− 6^	15.575
MOX	7581 (*gyrA*)	280G > A	− 3.340	− 3.694	2.221	< 10^− 6^	9.215

*Missense variant, unless specified; **Upstream Gene Variant; *RR* relative risk, *RIF* rifampicin, *INH* isoniazid, *EMB* ethambutol, *AMK* amikacin, *KAN* kanamycin, *ETH* ethionamide, *LEV* levofloxacin, *OFL* ofloxacin, *MOX* moxifloxacin.

## Discussion

With the increasing adoption of sequencing-based approaches for managing TB infections, generating substantial amounts of of *M. tuberculosis* data, there is a need to explore ML techniques for characterising the genetic mutations underlying drug resistance. In this study, we assess the predictive performance of ensemble tree-based approaches on a large-scale WGS and pDST dataset comprising over  35,000 MTBC isolates. We emphasise the importance of targeted approaches to enhance the interpretability and applicability of drug-resistance predictions, evaluating three different feature sets (F1–F3). Notably, despite its greater feature count, the F3 set, comprising common (MAF > 0.5%) genome-wide variants, did not outperform the F2 feature set, which focused on genomic variants in candidate drug-resistance genes. While drug resistance mutations outside known candidate genes may exist and be important for prediction, their rarity could lead to their exclusion from the genome-wide model. This resulted in reduced model dimensionality  and refined feature selection, though low-frequency genome-wide variants may be integrated into ML prediction in the future, particularly through aggregating features.

Importantly, the inclusion of co-occurring variants boosted the predictive performance for certain drugs, corroborating findings from previous studies^10,15^. For example, resistance to second-line drugs is often accompanied by resistance to first-line drugs due to treatment regimens. Consequently, mutations that cause RIF and INH resistance often rank highly in predictive models (see Data S1). While the presence of co-occurring mutations may reflect underlying biological processes, such as pre-resistance mutations increasing the risk of developing further resistance, they are not always causative^20^. Their inclusion can limit the interpretability and reliability of ML models, which is critical for understanding resistance-driving mutations in genomic surveillance and mitigating biases from dataset structures. To address these issues, we adopted a more targeted approach, using genomic variants from drug-specific genomic regions in our models. This strategy improved performance for EMB, AMK, and LEV, and did not have a detrimental impact on predictive performance, except for PZA and ETH. By concentrating on features with higher relevance, we reduced noise in the ML models. Our findings demonstrate the potential of ML and targeted approaches for drug-resistance diagnostics, particularly when combined with methods like targeted next-generation sequencing (e.g., Amplicon Sequencing (AMP-SEQ))^21^.

By supplementing the feature set with aggregated counts of rare mutations, we improved the predictive performance for INH, PZA, STM, CAP, MOX, ETH, and LEV. Aggregating rare variants allows us to consider their combined effects, which might otherwise be masked by more frequent variants. This approach is important for capturing emerging or under-sampled drug-resistant mutations in predictive models. Notably, pooled variants significantly enhanced sensitivity performance for PZA, as characterised by the high density of *pncA* mutations with additive small effects^22,23^. Similarly, sensitivity gains for ETH could be attributed to the pooling of high-impact variants in the *ethA* gene, supported by feature importance analysis. A multi-dimensional analysis of feature contributions further demonstrates the effect of pooling genomic information across models for multiple drugs, and highlights the capacity of such exploratory approaches to mine potentially novel resistance associations from large genomic datasets.

The observed performance improvements for INH, PZA, ETH and STM may have also stem from the combined impact of LOF mutations, which we investigated further through association analysis of their individual effects on drug-resistant phenotypes. We identified LOF mutations in *pncA* and *ethA*, including premature stop codons, deletions, and frameshifts, potentially preventing the activation of PZA and ETH into their active forms. Both drugs are pro-drugs that require activation by pyrazinamidase (*pncA*) and ethionamide monooxygenase (*ethA*), respectively^24,25^. Mutations in *mshA* were also found to be associated with INH and RIF resistance, and are implicated in the bio-activation pathway for ETH^26^. This insight suggests that LOF mutations may be a key resistance mechanism against pro-drugs in *M. tuberculosis*. In addition, multiple LOF mutations were observed in *Rv0678,* which encodes the transcriptional repressor of the Mmps5-MmpSL efflux pumps^27,28^. The overexpression of Mmps5-MmpSL confers cross-resistance to CLF and BDQ, highlighting its significance for targeted next-generation sequencing diagnostics^28^. Notably, LOF mutations were not exclusive to resistant isolates and were observed in some susceptible samples, potentially due to their association with low levels of resistance or pDSTs errors^5^. Two potential novel frameshift mutations in *Rv3861* (Ile27fs, STM) and *Rv1907c* (*Asp48fs*, INH) were observed across independent lineages and are absent from the current WHO mutation catalogue. Intriguingly, *Rv1907c* is part of the *katG* operon and has been associated with INH resistance^29^. After undergoing functional validation, the inclusion of such variants could help to increase the sensitivity of genetic-based DSTs and underlying mutation libraries.

Our best performing models are comparable to previously employed approaches on large-scale *M. tuberculosis* WGS data. While CNN models have demonstrated higher sensitivity^15^, likely due to their capacity for gene-level representation and retention of rare-variants, our increased sample size enhances the statistical power of our analyses. However, the underlying architecture and feature selection must also be considered to optimise model performance. A direct comparison using the same dataset would clarify whether improvements are due to sample size or model architecture. Nonetheless, comparing models across datasets can be challenging due to varying software requirements and input formats, alongside reproducibility issues in prior publications. Utilising standardised environments, such as Docker containers within ML-TB, could simplify this model comparison^16^. Further research will focus on optimising these models for practical deployment.

Post-hoc MIC-based analysis may further elucidate the effects of mutations on the severity of drug-resistance. For example, our MIC analysis revealed mutations in the nine-base pair segment of the *inhA* promoter region, previously associated with a binary resistance phenotype against INH, which also demonstrated evidence of homoplasy^30^. These mutations are linked to ‘extreme’ INH resistance phenotypes, where samples exhibit MICs over 100-fold higher than the resistance threshold. Similarly for ETH, a structural analogue of INH^31^, variants in the *inhA* promoter regions were also identified in significant association with the highest ordinal MIC phenotype. These findings may suggest a role for these promoter region variants in mediating the development of high-level INH and ETH resistance through affecting overexpression of the drug target. Although GWAS analysis have been applied to MIC data^32^, we focused on a targeted risk-ratio analysis of known loci across ordinal levels, which limited the number of statistical tests and allowed us to uncover significant enrichment within severe resistance phenotypes. Further research should examine variants associated with intermediate resistance profiles to identify similar effects that may not have been captured using GWAS methods^7^.  There is also potential to utilise MIC data for quantitative resistance predictions using ML approaches.

There are several limitations that could be addressed by future studies. Predictive performance for some drugs, such as CAP, remains limited and could benefit from a larger sample size or broader genomic coverage in feature sets. Additionally, confounding effects from population structure may affect our approaches, despite lineage inclusion; this could be mitigated through feature weighting or sampling approaches^12^. Such approaches could enhance model interpretability during training. Earlier studies recommended filtering out lineage-specific and synonymous mutations before model training to enhance the relevance of selected features^33^. However, both phylogenetic background and lineage-specific mutations have been linked to drug resistance^34^. Additionally, the role of synonymous mutations in drug resistance is gaining recognition, particularly concerning their potential to influence the translation of drug activators^35^. This underscores the evolving nature of feature selection, highlighting the need for ongoing research to adapt to these changes. Moreover, how features are represented in a model significantly impacts its performance, interpretability, and applicability. Following previous approaches, we used one-hot encoding for SNPs and indels, which may have led to limitations such as sparse matrices and reference bias, especially in datasets containing multiple lineages. Although alternative representations, like DNA sequences, could have been considered, we opted for one-hot encoding for its interpretatability and downstream deployment ease. Profiling tools like TB-Profiler, which conduct targeted variant calling for drug-resistance prediction^4^, could benefit from smoother integration with ML models utilising this format. Finally, while we have applied multivariate statistical approaches, future research should also explore the impacts of variant interactions, for example, on MIC phenotypes.

## Conclusion

In lieu of this future work, our study has demonstrated that the application of targeted, efficient, and interpretable ML approaches can lead to improved predictions of drug-resistant TB and diagnosis. These targeted ML models could be effectively combined with next-generation sequencing technologies, such as AMP-SEQ, to streamline the sequencing process and reduce resource requirements, making genetic-based DST approaches more accessible and scalable in low-resource settings^36^.

## Methods

### Sequencing data analysis

Illumina whole genome sequencing data (WGS) was sourced from public repositories/archives for 50,723 *M. tuberculosis* isolates^4^. Raw sequencing reads were aligned to the H37Rv reference genome (NCBI Reference Sequence: NC_000962.3) using the BWA-mem aligner (v0.7.17)^37^ following trimming with *Trimmomatic* (v0.39)^38^. SNPs and indels were identified on the aligned reads using *samtools*^39^ and joint genotyping was performed using GATK *GenotypeGVCFs* (v4.1.3.0) (gatk.broadinstitute.org)^39^. Monomorphic SNPs, variants in non-unique regions of the genome (e.g. *pe/ppe* genes), and variants with > 10% missing calls were excluded.

### Data pre-processing

To create predictive ML models for each drug, multiple feature matrices along with the corresponding phenotype/classification labels were built per drug for the relevant sample. Only the genomic loci with a non-major allele frequency (MAF) (recalculated after sub-setting samples for each drug) of greater than 0.1% were retained. This filtering was applied to control the number of input features for the ML model, mainly to limit computational complexity and time, and to prevent the model overfitting. The *bedtools* (*v2.31*)^40^ intersect tool was used to only retain genes associated with drug resistance and these were extracted from the TB-profiler list library (Table S6)^4^. For each sample, only the presence or absence of a variant (SNP, indel) at a given genomic locus was encoded, as 1 and 0, respectively, in a *numpy* matrix. Lineage information per isolate (up to the second degree, e.g. Lineage 4.1) was predicted using TB-Profiler^4^, and this data was appended to the feature matrices. The performance of ML models was generally observed across three different feature sets: (i) resistance-associated regions for the specific drug in question with MAF > 0.1% (F1; 56–143 variants; specific regions in Table S6); (ii) resistance-associated regions for any antibiotic drug profiled in the TB-Profiler database with MAF > 0.1% (18 drugs, 895–1428 mutations; Table S6) (F2), and variants across the whole genome (5603−10,487 variants after filtering for MAF > 0.5%) (F3) (Tables S9–S10). Rare variants, which are unlikely to have high feature importance, were excluded from the F1 and F2 datasets. However, the F1 dataset was supplemented with aggregated counts of rare variants predicted by SnpEff to have moderate-to-high functional impact (MAF < 0.1%), resulting in the F1 + dataset. This allowed for the combined effects of rare variants to be incorporated into the predictions. MAF cut-offs were determined using histograms to assess the distribution of rare variants, informing the thresholds for feature inclusion in each dataset (Fig. S11).

### Machine learning algorithms

The Random Forest (RF) and Gradient-boosted Tree (GBT) algorithms were used across the three feature sets (F1, F2 and F3) to train models to predict resistance to INH, RIF, EMB, PZA, STM, OFL, MOX, LEV, AMI, CAP, KAN, ETH and RFB. The *sklearn* Python library (v1.4.1)^41^ and the XG*Boost* Python API (1.7.6) were used for model implementation. All these approaches have been previously utilised in the context of training models for drug resistance prediction from WGS data^10,19^. Prior to training, the data was split into training (80%) and testing (20%) datasets. The majority (80%) of the data was used to train the model and 20% was used to calculate the hold-out performance for each tuned model. Hyperparameter tuning and classification threshold optimisation were performed using a stratified five-fold cross-validation on the training set using specified parameters and ranges through a Randomised Grid Search approach (Table S7)^41^. We report cross-validation and holdout-test performance results for all models, except for GBT + F3 (stratified 5-fold cross-validation results only). This was due to the high dimensionality of the input feature matrix, and thus only cross-validation results for the F3 feature set on default parameters are described (Table S3). A permutation feature importance algorithm was used to assess contributions of input variants to predictive performance.

### Loss-of-function association analysis

The *SNPEff* software (*version 5.1*)^42^ (*H37Rv* reference genome) was used for the functional annotation of variants found in the canonical drug-resistance associated regions (Table S6). Variants with a LOF annotation for at least one transcript were then selected. Associations between resistance and LOF variants were assessed using a Fisher’s exact test on a zero-padded contingency matrix and conditional odds ratios were calculated for all variants demonstrating at least five resistant isolates harbouring the alternate allele. P-values were adjusted using the Benjamini Hochberg method, and a threshold of 0.01 was applied to prioritise variants. An analysis of the lineage distribution of the putative LOF variants was performed post-hoc to account for population structure and validate robust markers linked to resistance.

### MIC phenotype analysis

Isolates with MIC phenotype values were categorised into four incremental levels (Susceptible, ‘Low’ resistance, ‘High’ resistance, and Resistant [‘Extreme’]) based on recommended ECOFF thresholds (Table S8). Variants in drug-specific resistance-associated regions with a MAF of 0.5% were selected. A proportional odds multivariate logistic regression model was fitted with all selected variants (*MASS* package in R^43^). Variants with regression coefficients > 1 and significant at the adjusted P-value (Benjamini Hochberg) threshold of 0.01 were further used as inputs into a multinomial regression model (*nnet* package in R^44^) to estimate relative-risk coefficients across each category (reference level = ‘High’).

## Electronic Supplementary Material

Below is the link to the electronic supplementary material.

Supplementary Material 1

Supplementary Material 2

## Data Availability

No new samples were sequenced for this study. The sample accession numbers, feature importance values, and relevant code for machine learning and statistical analysis is available in a dedicated GitHub repository: https://github.com/SSID08/TB-ML. Data S1 refers to the supplementary files held within this repository.
