# Supplementary material for: Leveraging large-scale Mycobacterium tuberculosis whole genome sequence data to characterise drug-resistant mutations using machine learning and statistical approaches
Source: Sci Rep. 2024 Nov 7;14:27091. doi: 10.1038/s41598-024-77947-w (PMC11544221; doi:10.1038/s41598-024-77947-w)
Supplement: Supplementary file 1 — Supplementary Material 1 [file 41598_2024_77947_MOESM1_ESM.pdf]

Leveraging large-scale *Mycobacterium tuberculosis* whole genome sequence data to characterise drug-resistant mutations using machine learning and statistical approaches

Siddharth Sanjay Pruthi[1,2], Nina Billows[1], Joseph Thorpe[1], Susana Campino[1], Jody E. Phelan[1,*], Fady Mohareb[2,*], Taane G. Clark[1,3,*,]**

[1]Faculty of Infectious and Tropical Diseases, London School of Hygiene & Tropical Medicine, WC1E 7HT London, UK
[2]School of Water, Energy and Environment, Cranfield University, Bedford, UK.
[3]Faculty of Epidemiology and Population Health, London School of Hygiene & Tropical Medicine, WC1E 7HT London, UK

* Joint authors

** Corresponding author: Prof. Taane G. Clark, Department of Infection Biology, Faculty of Infectious and Tropical Diseases, London School of Hygiene & Tropical Medicine, Keppel Street, London, UK

Supplementary Figures

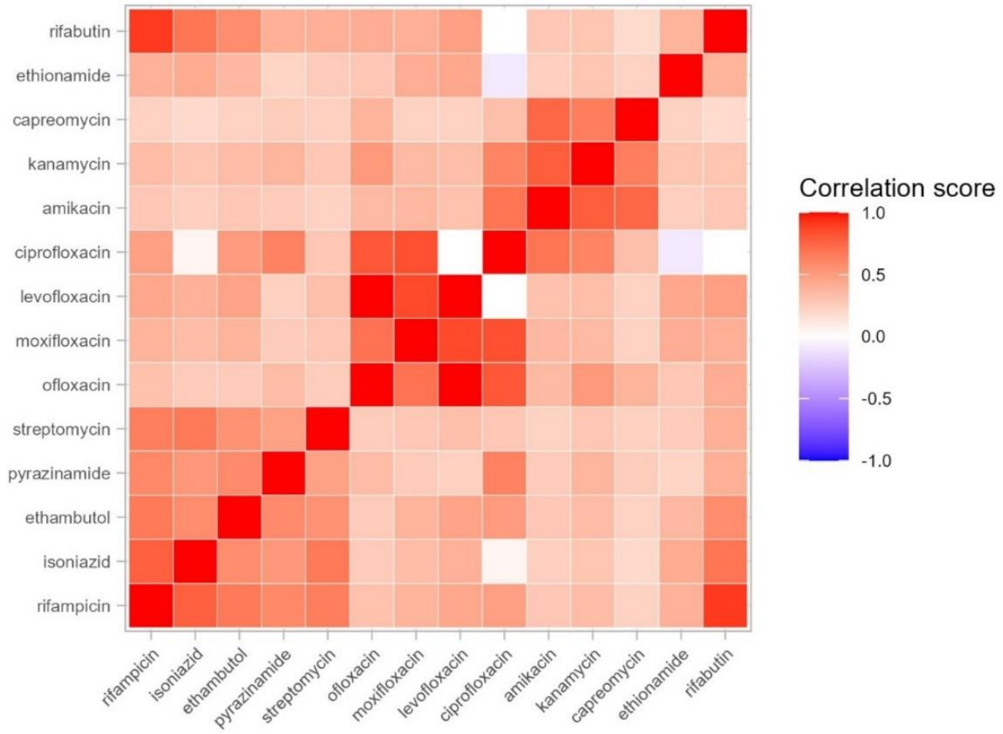

Figure S1. Pearson correlation between resistance phenotypes across 14 drugs.

Pearson correlation coefficients were calculated between MIC values for 14 drugs. Correlation scores are coloured on the heatmap to indicate strong resistance co-occurrence between drugs.

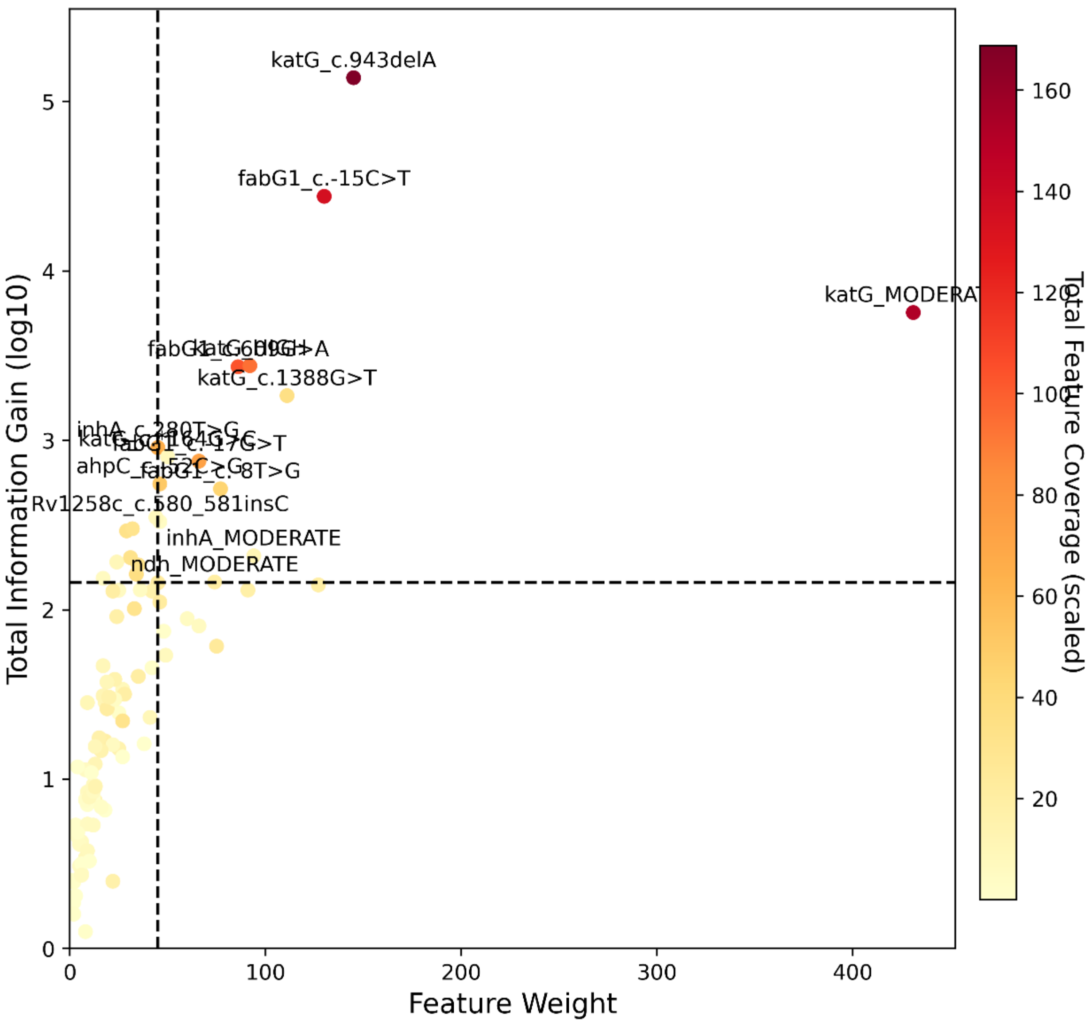

Figure S2. Feature importance and feature coverage for isoniazid (INH)

Representation of feature importance values across *Information Gain*, *Feature Coverage* (average number of instances affected by the feature), and *Feature Weight* (number of times the feature appears across all trees) in models (GBT-F1+ counts) for INH. Values labelled if in the 20th-percent-tile across both *Information Gain* and *Feature Weight.*

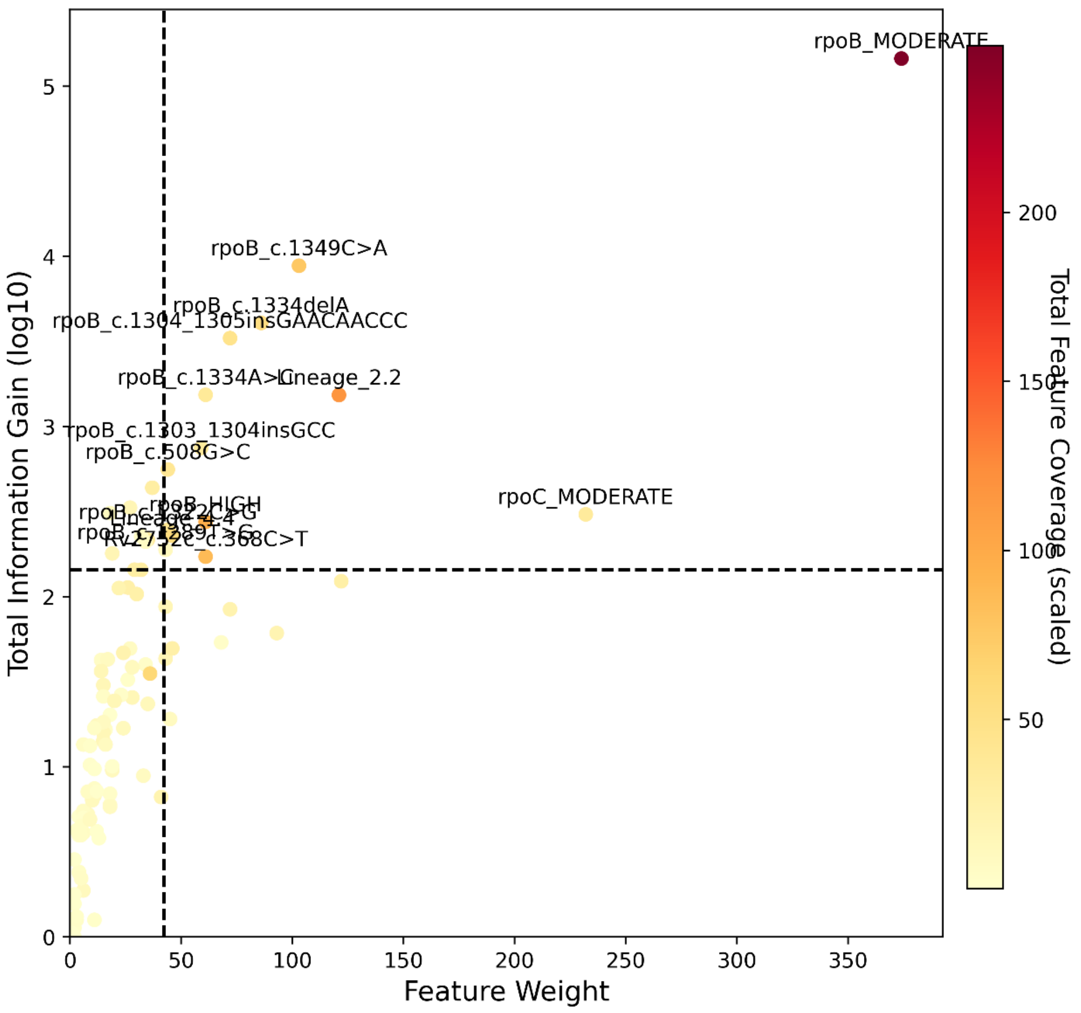

Figure S3. Feature importance and feature coverage for rifampicin (RIF)

Representation of feature importance values across *Information Gain*, *Feature Coverage* (average number of instances affected by the feature), and *Feature Weight* (number of times the feature appears across all trees) in models (GBT-F1+ counts) for RIF. Values labelled if in the 20th-percent-tile across both *Information Gain* and *Feature Weight.*

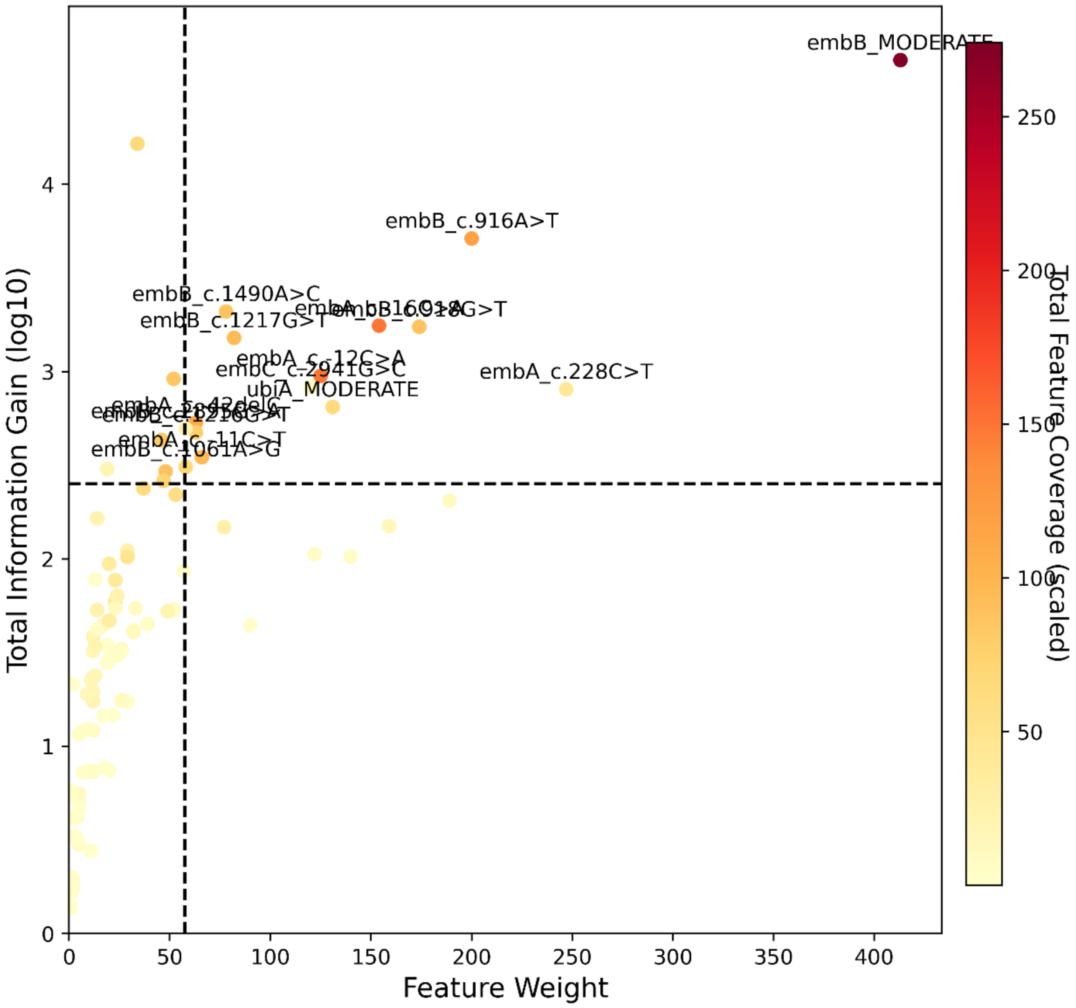

Figure S4. Feature importance and feature coverage for ethambutol (EMB)

Representation of feature importance values across *Information Gain*, *Feature Coverage* (average number of instances affected by the feature), and *Feature Weight* (number of times the feature appears across all trees) in models (GBT-F1+ counts) for EMB. Values labelled if in the 20th-percent-tile across both *Information Gain* and *Feature Weight.*

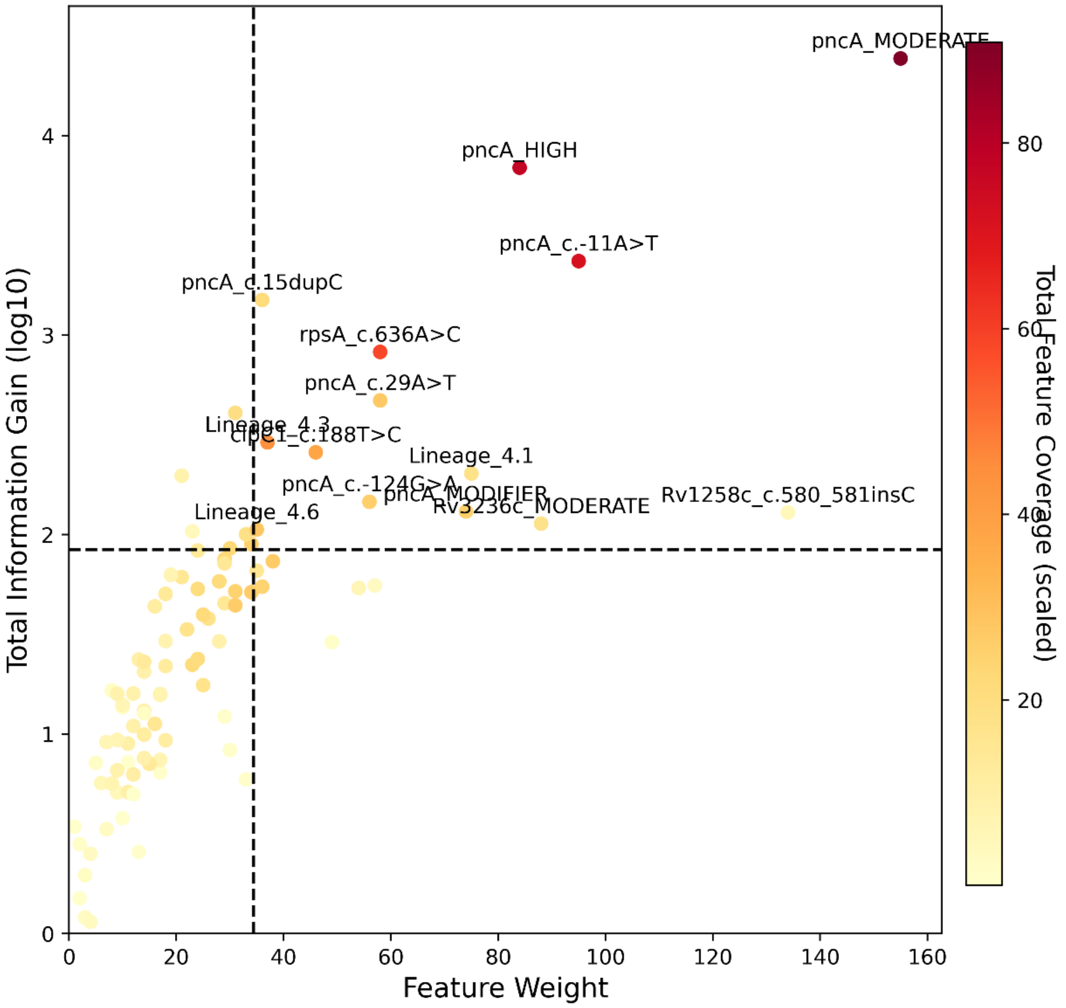

Figure S5. Feature importance and feature coverage for pyrazinamide (PZA)

Representation of feature importance values across *Information Gain*, *Feature Coverage* (average number of instances affected by the feature), and *Feature Weight* (number of times the feature appears across all trees) in models (GBT-F1+ counts) for PZA. Values labelled if in the 20th-percent-tile across both *Information Gain* and *Feature Weight.*

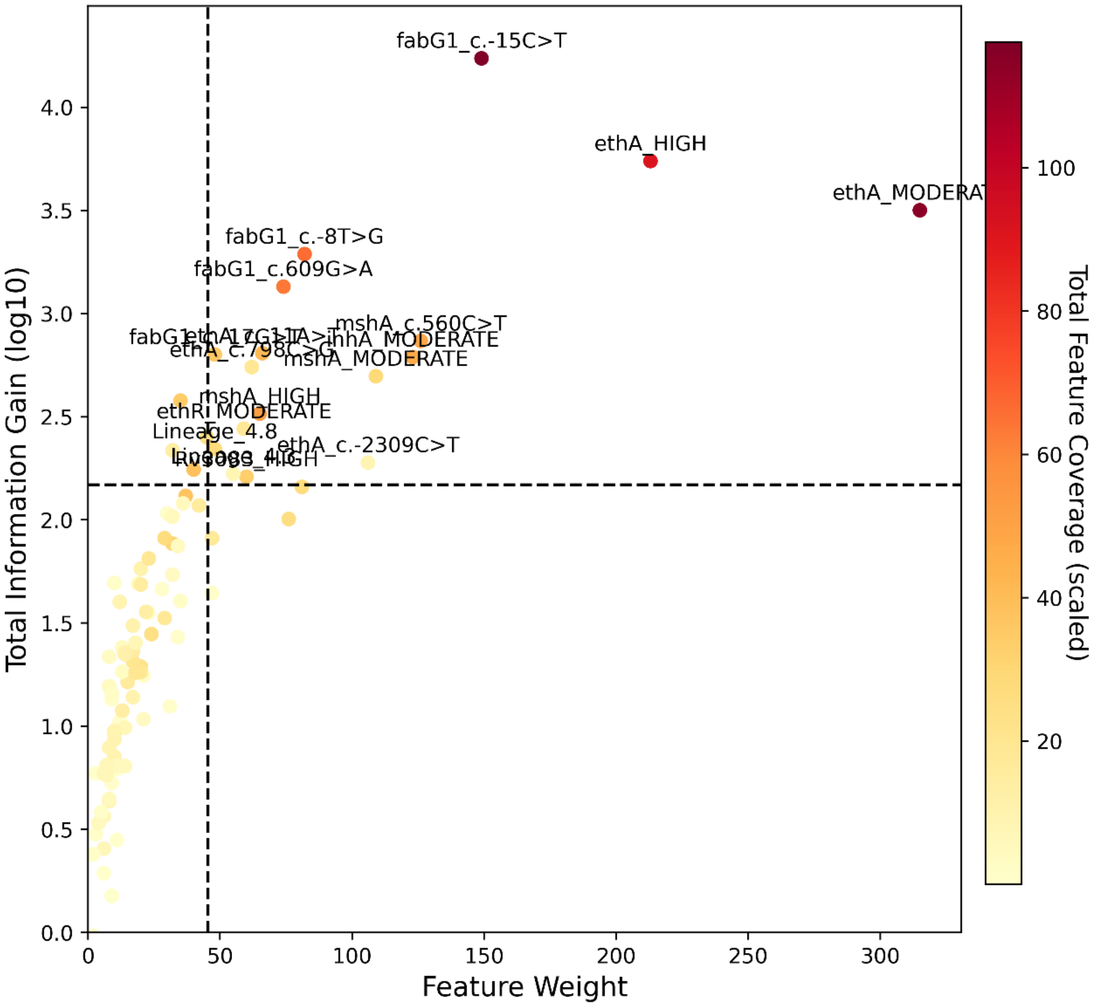

Figure S6. Feature importance and feature coverage for ethionamide (ETH)

Representation of feature importance values across *Information Gain*, *Feature Coverage* (average number of instances affected by the feature), and *Feature Weight* (number of times the feature appears across all trees) in models (GBT-F1+ counts) for ETH. Values labelled if in the 20th-percent-tile across both *Information Gain* and *Feature Weight.*

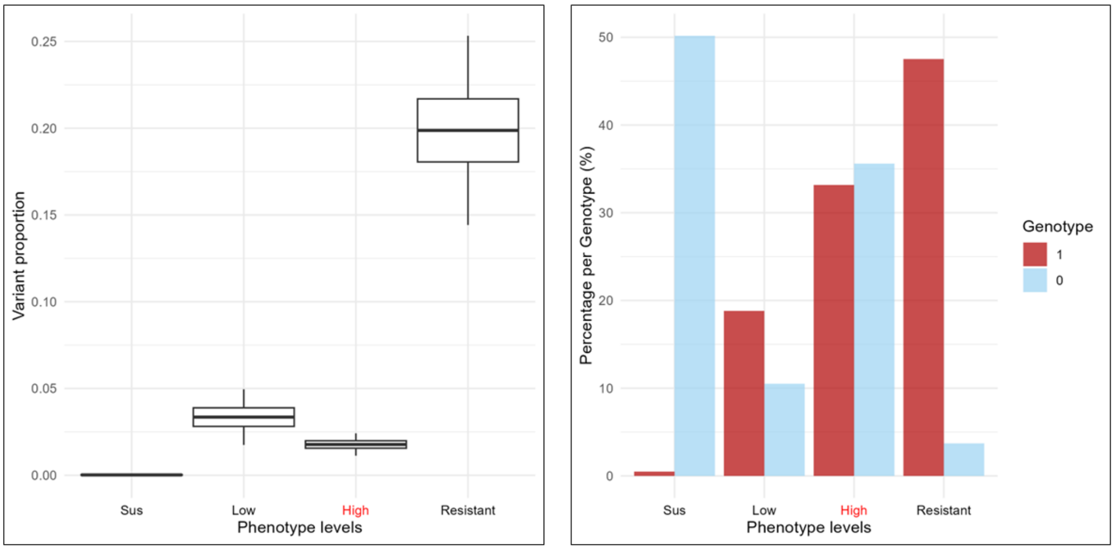

Figure S7. Distribution of *inhA -c.779/fabG1* -17G>T across isoniazid (INH) minimum inhibitory concentration (MIC) phenotypes.

Allele frequencies (left) and genotype distribution (right) of variant across ordinal MIC phenotypes for INH.

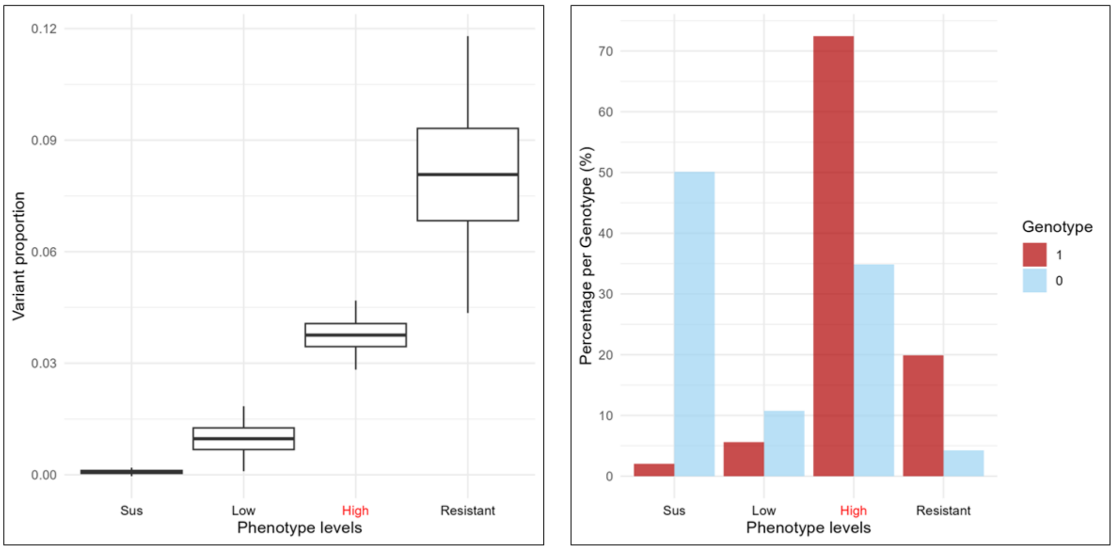

Figure S8. Distribution of *inhA -c.770*/*fabG1* -8T>C/G across isoniazid (INH) minimum inhibitory concentration (MIC) phenotypes

Allele frequencies (left) and genotype distribution (right) of variant across ordinal MIC phenotypes for INH.

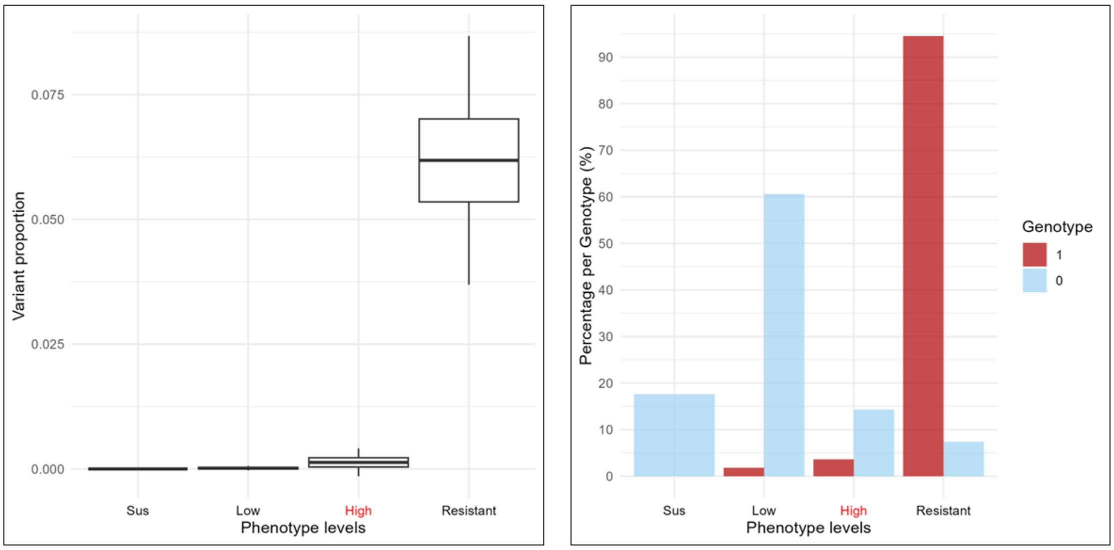

Figure S9. Distribution of *inhA c.*62T>C across ethionamide (ETH) minimum inhibitory concentration (MIC) phenotypes

Allele frequencies (left) and genotype distribution (right) of variant across ordinal MIC phenotypes for ETH.

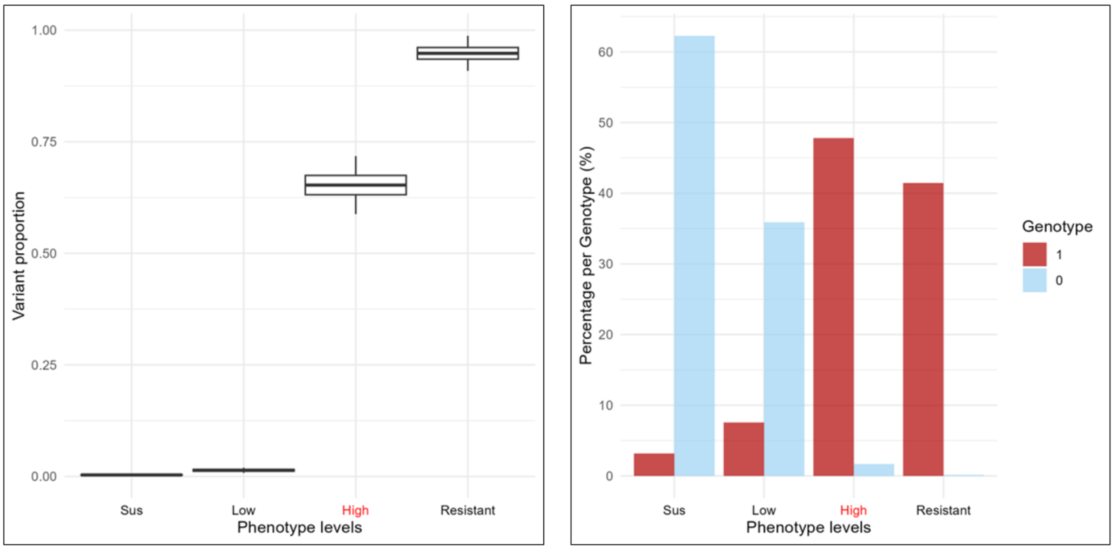

Figure S10. Distribution of *Rv1313c* c.-3471T>C across amikacin (AMK) minimum inhibitory concentration (MIC) phenotypes

Allele frequencies (left) and genotype distribution (right) of variants across ordinal MIC phenotypes for AMK.

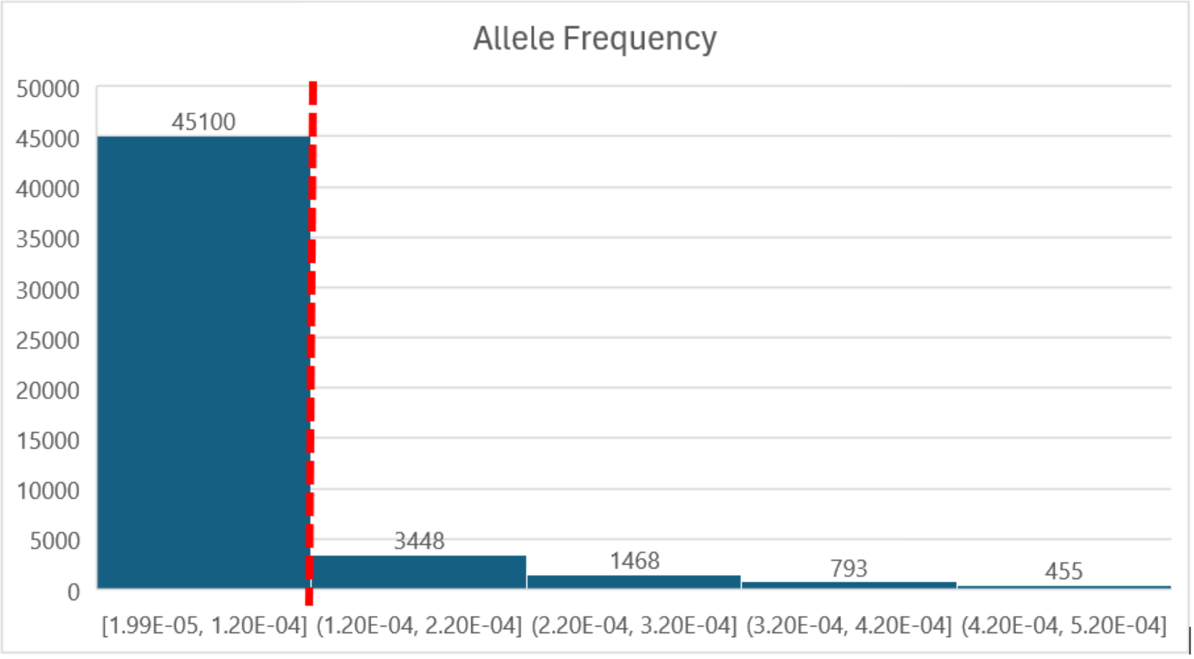

Figure S11. Allele frequency histogram of variants in drug-resistance genes

Allele frequencies of variants in drug-resistance genes with non-major allele frequency (MAF <0.005) are shown to highlight how the MAF threshold of 0.1% was determined. The dashed line indicates a threshold of 0.0001.
